# Rare case of acinar cell carcinoma with multiple lesions in the pancreas

**DOI:** 10.1002/jgh3.12380

**Published:** 2020-06-29

**Authors:** Yuki Ikeda, Makoto Yoshida, Kazuma Ishikawa, Tomohiro Kubo, Yasutoshi Kimura, Tadashi Hasegawa, Koji Miyanishi, Junji Kato

**Affiliations:** ^1^ Department of Medical Oncology Sapporo Medical University Sapporo Japan; ^2^ Department of Surgery, Surgical Oncology and Science Sapporo Medical University Sapporo Japan; ^3^ Department of Surgical Pathology Sapporo Medical University Sapporo Japan

**Keywords:** acinar cell carcinoma, metachronous, pancreatic cancer, synchronous

## Abstract

We present the first case of pancreatic acinar cell carcinoma (PACC) with multiple lesions. A 55‐year‐old man with a pancretic tail mass on abdominal computed tomography (CT) was admitted to our hospital. Endoscopic ultrasound (EUS) showed a hypoechoic mass, and EUS‐guided fine‐needle aspiration (EUS‐FNA) revealed the mass to be PACC. The patient underwent distal pancreatectomy, and two masses were identified in the pancreatic tail and body. Histologically, both masses had tumor cells similar to acinar cells and were positive for BCL‐10. The patient was thus diagnosed with synchronous PACC. Ten months after the surgery, abdominal CT revealed a mass in the remnant pancreas. EUS showed a hypoechoic mass, and EUS‐FNA determined it to be PACC. The patient underwent total remnant pancreatectomy. The histological imaging results were similar to those of the first resection. Finally, the patient was diagnosed with synchronous and metachronous PACC. The possibility of multiple occurrences in the pancreas should be considered with PACC.

## Graphical abstract

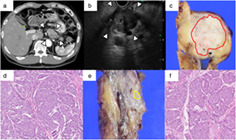

We present the case of a 55‐year‐old man who had pancreatic acinar cell carcinoma (PACC) and underwent distal pancreatectomy. Two masses were identified in the pancreatic tail and body and were diagnosed as synchronous PACC. Ten months after the surgery, a mass was found in the remnant pancreas, and total remnant pancreatectomy was performed, resulting in the diagnosis of synchronous and metachronous PACC.

## Introduction

Acinar cell carcinoma (ACC) of the pancreas is relatively rare, accounting for approximately 1% of all pancreatic neoplasms.^1^ ACC exhibits a wide variety of imaging findings, often grows expansively, and easily develops in the main pancreatic duct and in the portal vein compared with pancreatic ductal adenocarcinoma (PDAC).^2^ Although there are some reports of multiple PDAC, there are no reports of multiple ACC in pancreas.

Here, we report a rare case of multiple ACC that occurred synchronously and metachronously in the pancreas.

## Case report

**Figure 1:**
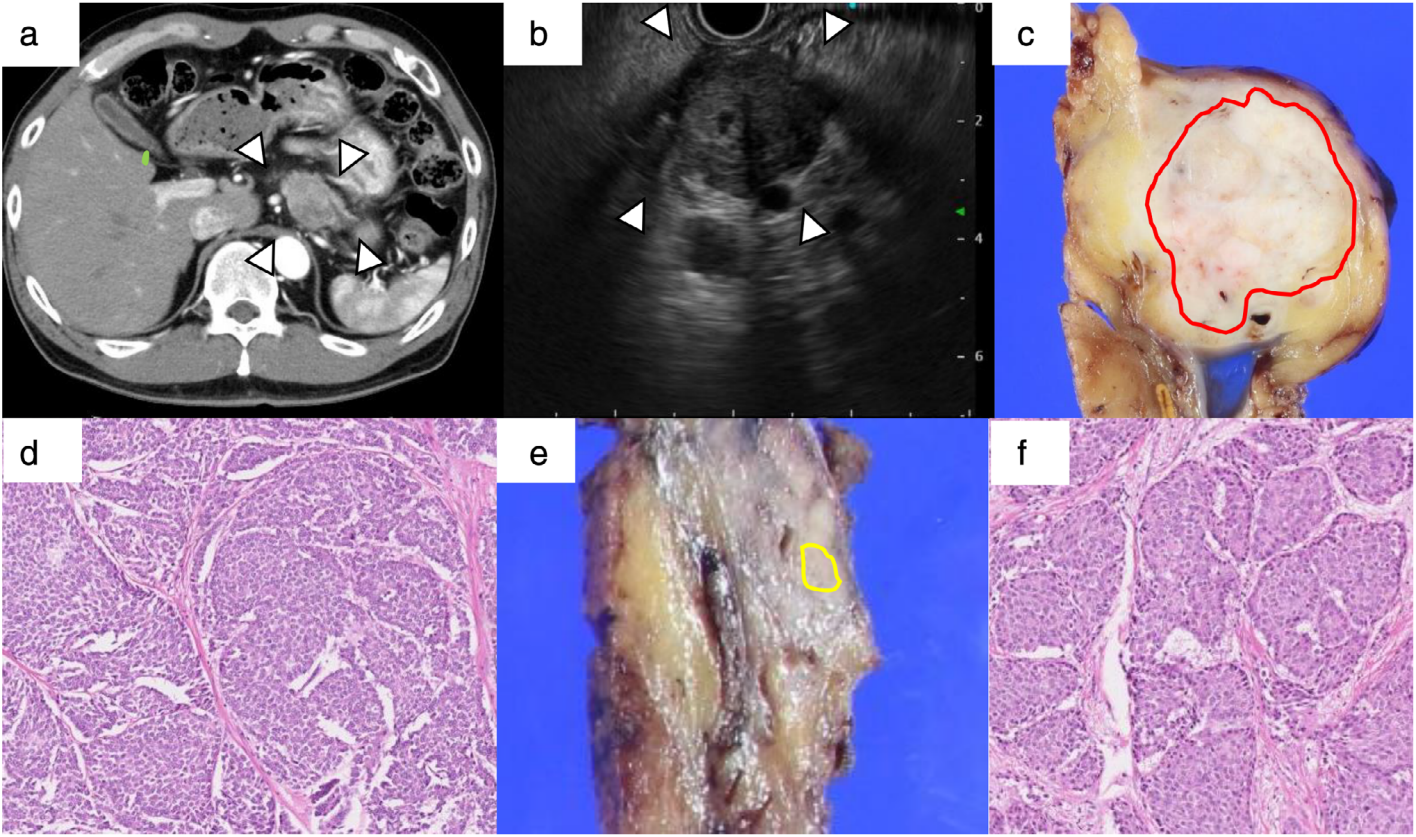
Contrast‐enhanced abdominal computed tomography (a) demonstrated a 40‐mm heterogeneous tumor at the tail of the pancreas (arrow head); endoscopic ultrasound (b) revealed a hypoechoic mass at the tail of the pancreas (arrow head). The cut surface of the resected specimen showed two white–grayish masses in the pancreatic tail (c, red line) and pancreatic body (e, yellow line). Hematoxylin and eosin staining revealed tumor cells similar to acinar cells in the pancreatic tail (d) and body (f).

A 55‐year‐old man with a pancreatic mass on contrast‐enhanced computed tomography (CT) performed due to acute alcoholic pancreatitis was admitted to our hospital. His serum cancer antigen 19‐9 level was mildly elevated at 54 U/mL (normal range, 0–37 U/mL). Abdominal CT revealed a 40‐mm heterogenous mass with a clear boundary in the tail of the pancreas (Fig. 1a). Pancreatic cancer, neuroendocrine neoplasm (NEN), solid pseudopapillary neoplasm (SPN), and inflammatory change were considered in the differential diagnosis. Endoscopic ultrasound (EUS) showed a heterogenous hypoechoic mass with partial necrosis (Fig. 1b). Histological examination of an EUS‐guided fine‐needle aspiration (EUS‐FNA) specimen revealed the presence of tumor cells with round nuclei and eosinophilic vesicles. Immunohistological staining was positive for BCL‐10. The patient underwent distal pancreatectomy. The resected specimen showed two white–grayish masses in the pancreatic tail (Fig. 1c) and pancreatic body (Fig. 1e), and normal pancreatic tissue was found between the two tumors. Histologically, tumor cells similar to acinar cells with round nuclei, eosinophilic vesicles, and a solid growth pattern were found in the two lesions (Fig. 1d,f). Both lesions were positive for BCL‐10 and were diagnosed as synchronous pancreatic acinar cell carcinoma (PACC). S‐1 was administered as postoperative chemotherapy.

Ten months after the surgery, abdominal CT demonstrated a 20‐mm hypovascular mass in the remnant pancreas, and EUS‐FNA revealed the presence of PACC. The patient underwent total remnant pancreatectomy. The histological features of the tumor in the remnant pancreas resembled the PACC histology of the first resected mass. No tumor cells were found in the resection margin. Finally, the patient was diagnosed with synchronous and metachronous PACC.

## Discussion

ACC of the pancreas is relatively rare, accounting for about 1% of all pancreatic neoplasms.^1^, ^3^ Compared with PDAC, ACC exhibits a wide variety of imaging findings, often grows expansively, and easily develops in the main pancreatic duct and portal vein.^2^, ^4^ In addition, cystic degneration, bleeding, and calcification are often observed within PACC.^5^ It is difficult to diagnose PACC on the basis of radiological findings alone, and its differential diagnosis includes PDAC, NEN, SPN, and inflammatory change. Although preoperative histological analysis is important, immunostaining can be performed on FNA tissue, allowing PACC diagnosis.^6^ The 5‐year survival rate of PACC after surgical resection is considered to be better than that of PDAC.^1^ The prognosis is still poor for postoperative relapse or unresectable patients. The most common metastatic site is the liver, similar to PDAC.^7^ Although there are some reports of multiple PDAC, there have been no reports of multiple PACC. Thus, this is the first report to describe synchronous and metachronous PACC diagnosed by EUS‐FNA. The possibility of multiple occurrences in the pancreas should be considered with PACC.

## References

[1] Kitagami H , Kondo S , Hirano S , Kawakami H , Egawa S , Tanaka M . Acinar cell carcinoma of the pancreas: clinical analysis of 115 patients from Pancreatic Cancer Registry of Japan Pancreas Society. Pancreas. 2007; 35: 42–6. 17575544 10.1097/mpa.0b013e31804bfbd3

[2] Basturk O , Zamboni G , Klimstra DS *et al* Intraductal and papillary variants of acinar cell carcinomas: a new addition to the challenging differential diagnosis of intraductal neoplasms. Am. J. Surg. Pathol. 2007; 31: 363–70. 17325477 10.1097/01.pas.0000213376.09795.9f

[3] Wang Y , Wang S , Zhou X *et al* Acicar cell carcinoma: a report of 19 cases with a brief review of the literature. World J. Surg. Oncol. 2016; 14: 172. 27352960 10.1186/s12957-016-0919-0 PMC4924290

[4] Hashimoto M , Matsuda M , Watanabe G *et al* Acinar cell carcinoma of the pancreas with intraductal growth: report of a case. Pancreas. 2003; 26: 306–8. 12657959 10.1097/00006676-200304000-00016

[5] Talti S , Mortele KJ , Levy AD *et al* CT and MRI featres of pure acinar cell carcinoma of the pancreas in adults. AJR Am. J. Roentgenol. 2005; 184: 511–19. 15671372 10.2214/ajr.184.2.01840511

[6] Ohno A , Sato Y , Nakamura E *et al* Cytological findings and BCL10 expression in pancreatic acinar cell carcinoma: a case report. Diagn. Cytopathol. 2017; 45: 247–51. 27860444 10.1002/dc.23634

[7] Chaudhary P . Acinar cell carcinoma of the pancreas: a literature review and update. Indian J. Surg. 2015; 77: 226–31. 26246707 10.1007/s12262-014-1049-y PMC4522262

